# Combined HMOs and α-MFGM Ameliorate LPS-Induced Intestinal Mucosal and Systemic Immune Dysfunction via Modulation of Gut Microbiota

**DOI:** 10.3390/nu18183057

**Published:** 2026-09-18

**Authors:** Jianwei Wu, Zhenye Shi, Jiayue Li, Chuqi Jiang, Bailiang Li, Song Wang

**Affiliations:** 1 Key Laboratory of Dairy Science, Ministry of Education, Northeast Agricultural University, Harbin 150030, China; 2 Food College, Northeast Agricultural University, Harbin 150030, China; 3 Nestlé Development Unit Nutrition, Jing’an District, Shanghai 200041, China

**Keywords:** human milk oligosaccharides (HMOs), α-milk fat globule membrane (α-MFGM), combined effect, intestinal mucosal immunity, lipopolysaccharide (LPS), gut microbiota

## Abstract

Background: The intestine functions as the body’s primary immune barrier, playing a vital role in host defense against pathogens. Human milk oligosaccharides (HMOs: 2′-fucosyllactose and lacto-N-neotetraose) and α-milk fat globule membrane (α-MFGM) are key bioactive components in human milk known to regulate intestinal immunity. Although their individual benefits have been reported, the potential combined effects of their combined use on intestinal mucosal and systemic immune function remain poorly understood. Methods: To address this, we established a lipopolysaccharide (LPS)-induced intestinal injury model in BALB/c mice. The animals were randomly divided into five groups: normal control, LPS model control, α-MFGM alone, HMOs alone (2′-FL + LNnT), and combined α-MFGM + HMOs intervention. After 15 days of oral gavage, colonic histopathology, intestinal barrier integrity, immune organ indices, serum inflammatory cytokine levels, immune cell subsets, and gut microbiota composition were comprehensively evaluated. Results: Combined supplementation with HMOs and α-MFGM effectively alleviated colonic pathological damage, increased goblet cell numbers and mucus secretion, enhanced sIgA expression, and reduced serum DAO and D-lactate levels, indicating restored intestinal barrier function. It also balanced spleen and thymus indices, suppressed pro-inflammatory TNF-α/IL-6 production, elevated anti-inflammatory IL-10/IL-22 levels, and corrected the LPS-induced Th1/Th2 and Th17/Treg imbalances. Moreover, the combined intervention remodeled the gut microbiota by enriching beneficial *Lactobacillus* and *Roseburia* while decreasing the abundance of *Prevotella* abundance, with functional predictions pointing to enhanced butanoate metabolism. Conclusions: These findings demonstrate that HMOs and α-MFGM exert combined protective effects against LPS-induced intestinal immune dysfunction through modulation of the gut microbiota and its metabolic products, providing a scientific foundation for optimizing early-life nutritional strategies.

## 1. Introduction

The intestine represents the largest interface between the external environment and the internal milieu of the human body. It functions as an immune barrier, preventing pathogen entry and subsequent disease [1], and is recognized as the body’s largest immune organ [2]. The development of the intestinal immune system commences in utero and continues to mature through early childhood. Promoting the development of this system is crucial for reducing susceptibility to infections and diseases [3]. The intestinal mucosal immune system constitutes the core component of intestinal immunity. Through its constant interaction with the external environment (e.g., food, microorganisms, toxins), it performs vital immune defense functions [4]. The intestinal mucosa serves not only as the first line of defense against pathogenic microbes but also bears the critical task of maintaining immune tolerance to prevent excessive immune responses that could damage host tissues [5].This system comprises immune cells, immune molecules, the gut microbiota, and their intricate interactions, forming a complex immune network [6].

Human milk is the optimal source of nutrition for infants during lactation. While providing essential nutrients, it also plays a crucial role in immune protection [7]. Human milk oligosaccharides (HMOs), constituting the third largest solid component in human milk after lactose and lipids [8], are a class of complex sugar molecules with remarkable structural diversity. This diversity and their associated functionalities endow HMOs with biological importance in immune modulation, the establishment and balance of the gut microbiota, and the maintenance of intestinal barrier function [9]. Among them, 2′-fucosyllactose (2′-FL) and lacto-N-neotetraose (LNnT) are the two most commonly studied HMOs [10].

2′-FL is the most abundant fucosylated oligosaccharide, accounting for over 30% of total HMOs [11]. Existing research indicates that 2′-FL can modulate intestinal immune function by promoting the colonization of beneficial bacteria, such as bifidobacteria, in the gut [12]. As a neutral HMO, LNnT has also been shown to be beneficial in promoting the development of the infant intestinal immune system and the maturation of the intestinal barrier [13]. Recent studies have demonstrated that the synergistic action of 2′-FL and LNnT is more effective than individual components in promoting functional differentiation of the microbiota and optimizing its structure [14]. This synergy helps suppress pro-inflammatory-factor-mediated intestinal barrier damage, maintain epithelial integrity, and improve immune function through fermentation metabolites, namely short-chain fatty acids (SCFAs) [15].

Lipids in human milk exist in the form of milk fat globules [16]. These consist of a triglyceride core surrounded by a tri-layer membrane structure composed of phospholipids, sphingolipids, sterols, and proteins, known as the milk fat globule membrane (MFGM). The MFGM contains over 200 bioactive components, providing not only nutritional support but also playing a significant role in immunomodulation [17]. Studies have shown that MFGM can enhance host immune defense by strengthening intestinal immune function, improving the intestinal barrier, and promoting the balance of the gut microbiota [18].

The immunomodulatory effects of MFGM have prompted researchers to attempt to reconstruct a similar lipid structure in infant formula [19]. Alpha-Milk Fat Globule Membrane (α-MFGM) is a specialized nutritional component developed in this context. It is prepared by combining alpha-lactalbumin with the milk fat globule membrane through ultrafiltration and diafiltration, enabling infant formula to form a unique lipid globule structure with a nutrient profile closer to that of human milk in both content and proportion. A clinical trial conducted in Chinese infants demonstrated that formula supplemented with α-MFGM provides benefits in immunity, growth, gut health, and quality of life, similar to those observed in breastfed infants [20].

Current research indicates that bioactive components in human milk (HMOs and MFGM) play key roles in establishing the infant gut ecosystem and supporting growth and development [21]. α-MFGM is rich in various functional lipids and proteins and has been proven to promote intestinal barrier function, modulate immune responses, and influence gut microbiota composition. Meanwhile, HMOs, as human-milk-specific prebiotics, can selectively promote the proliferation of beneficial bacteria (e.g., bifidobacteria), thereby optimizing gut microecological balance [12]. Some studies have found combined interactions between HMOs and components within MFGM. An in vitro study showed that a combination of 3′-sialyllactose (3′-SL) and osteopontin (OPN) exhibited combined antiviral activity against influenza virus [22]. Similarly, research has indicated that a Maillard conjugate of 2′-FL and lactoferrin hydrolysates can modulate gut microbiome composition and health-promoting activity in mice [23]. However, it remains unclear whether combined use of HMOs and α-MFGM confers additional benefits beyond those of each component alone.

We hypothesized that co-administration of HMOs and α-MFGM would exert enhanced protective effects against LPS-induced intestinal injury via modulation of the gut microbiota. To test this hypothesis, we established a mouse model of intestinal mucosal immune injury via intraperitoneal injection of lipopolysaccharide (LPS) and systematically evaluated the individual and combined effects of HMOs and α-MFGM on intestinal barrier function, immune cell subsets, and gut microbiota composition.

## 2. Materials and Methods

### 2.1. Experimental Methods

#### 2.1.1. Animal

Thirty healthy BALB/c mice (specific pathogen-free (SPF) grade, male, 6 weeks old, 18–20 g) were purchased from Liaoning Changsheng Biotechnology Co., Ltd., Shenyang, China. The experimental environment was set to a 12-h light/dark cycle, with temperature maintained at 22 ± 2 °C and humidity at 45 ± 5%. After a 7-day acclimatization period, the mice were randomly divided into 5 groups (*n* = 6 per group) for the experiment. The sample size was determined by an a priori power analysis using G*Power software (version 3.1.9.7). The diarrhea score was predefined as the primary outcome measure. Based on the effect size observed in our own data (F(4, 25) = 42.25, *p* < 0.001, η^2^ = 0.871, Cohen’s f = 2.598), with α = 0.05 and power (1 − β) = 0.80, the analysis indicated that a minimum of 3 animals per group was required. To account for potential attrition (e.g., mortality after LPS injection, gavage failure, or sample loss) and biological variability, we adopted n = 6 per group for diarrhea and DAI scoring, and *n* = 3 per group for all other analyses. At these sample sizes, the achieved power exceeded 0.99. This study was approved by the Animal Ethics Committee of Northeast Agricultural University (ethical approval code: NEAUEC20240489).

#### 2.1.2. Experimental Design

**Figure 1:**
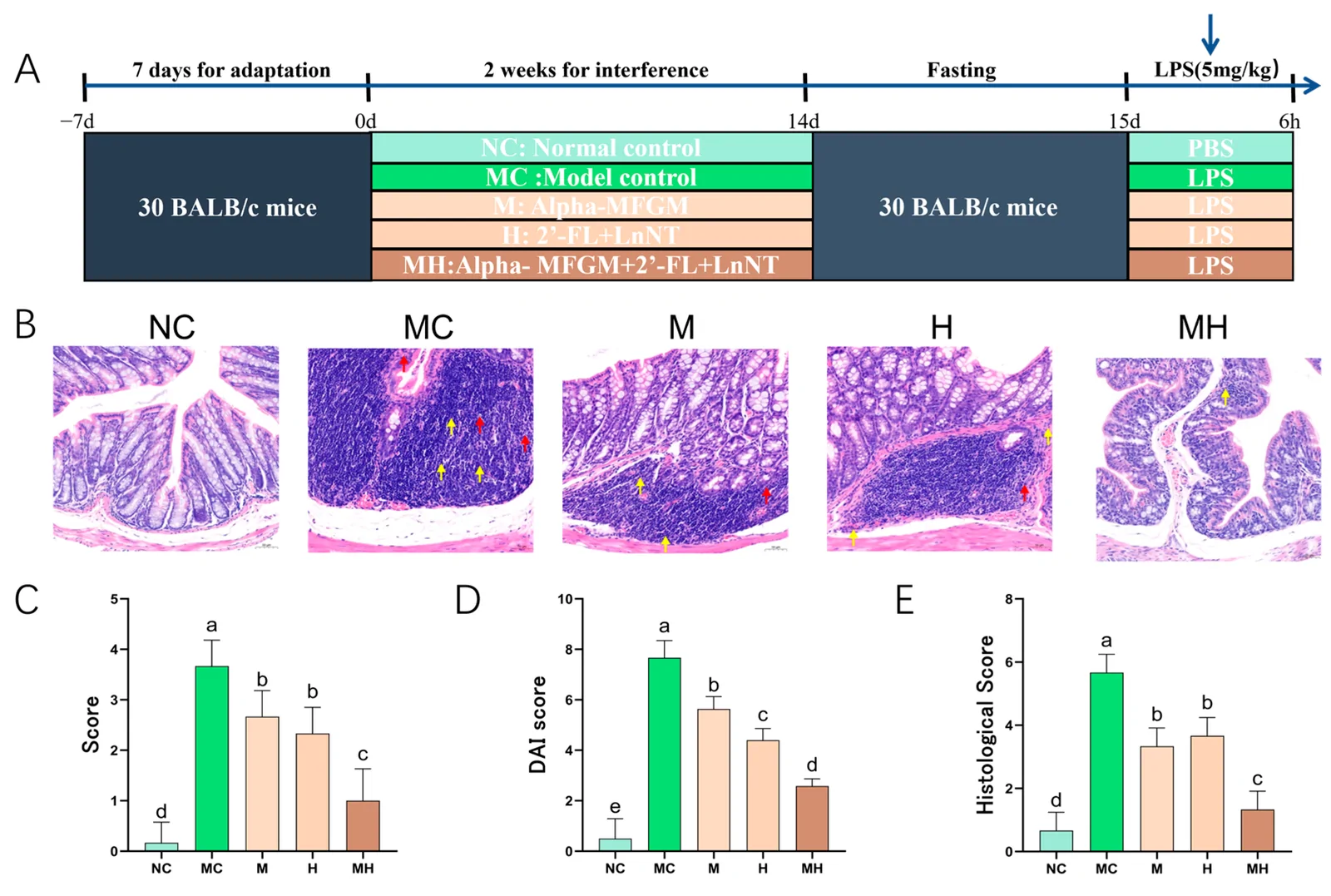
(**A**) Animal model experimental design. (**B**) HE staining (200× magnification). Yellow arrows indicate inflammatory cell infiltration; red arrows indicate cell necrosis. (**C**) Mouse diarrhea scores. (**D**) Disease Activity Index (DAI). (**E**) Colon histopathological scores. All data are presented as the mean ± SD (*n* = 3). Values with different superscript letters are significantly different at *p* < 0.05.

As shown in Figure 1A, dosages for the mice were calculated based on body surface area (BSA). The daily intake doses were set at 3697.222 mg/kg for α-MFGM, 1018.518 mg/kg for 2′-FL, and 509.259 mg/kg for LNnT. The experimental groups were as follows: Normal Control group (NC group, daily gavage with 0.2 mL PBS solution), LPS Model Control group (MC group, daily gavage with 0.2 mL PBS solution), α-MFGM group (M group, daily gavage with 0.2 mL α-MFGM solution), 2′-FL + LNnT group (H group, daily gavage with 0.2 mL mixed solution of 2′-FL and LNnT), and α-MFGM + 2′-FL + LNnT group (MH group, daily gavage with 0.2 mL mixed solution of α-MFGM, 2′-FL, and LNnT).

At the end of the 15-day gavage period, all groups except the NC group received an intraperitoneal injection of 0.2 mL LPS solution at a dose of 5 mg/kg. The NC group received an intraperitoneal injection of 0.2 mL PBS solution at the same nominal dose (5 mg/kg). All mice were observed for an additional 6 h and then euthanized by CO_2_ inhalation followed by cervical dislocation. All efforts were made to minimize suffering throughout the experiment. Serum, colon, spleen, thymus, and intestinal contents were collected for subsequent analysis.

All 6 mice per group were included in diarrhea and DAI scoring. For serum ELISA, histopathological analyses, and flow cytometry, 3 mice per group were randomly selected using a random number table.

### 2.2. Hart and Dobb Diarrhea Scale and Disease Activity Index Scoring

The severity of diarrhea in mice was assessed daily using the Hart and Dobb diarrhea scoring scale (Table 1), with fecal appearance as the primary criterion. The Disease Activity Index (DAI) was calculated by combining scores from three aspects: body weight change, stool consistency, and fecal blood (Table 2). Both indices were used together to reflect the degree of intestinal inflammation and the efficacy of the interventions.

### 2.3. Hematoxylin-Eosin (HE) Staining and Alcian Blue-Periodic Acid-Schiff (AB-PAS) Staining

**HE Staining:** After dissection, approximately 1 cm segments of colon were collected from the mice, cleared of intestinal contents, and immediately fixed in 4% paraformaldehyde (Sigma-Aldrich, St. Louis, MO, USA) for 24 h. The tissues were then dehydrated through a graded ethanol series (70–100%) (Sinopharm Chemical Reagent Co., Ltd., Shanghai, China), cleared in xylene (Sinopharm Chemical Reagent Co., Ltd., Shanghai, China), and embedded in paraffin wax (Leica Biosystems, Wetzlar, Germany) at 60 °C for 2 h. Continuous sections of 4 μm thickness were cut using a rotary microtome (Leica RM2016, Shanghai Leica Instrument Co., Ltd., Shanghai, China). The sections were baked at 60 °C for 1 h, followed by deparaffinization and rehydration. This process involved immersion in xylene I and II (10 min each) and a reverse graded ethanol series (100–70%, 5 min each), concluding with a rinse in distilled water. HE staining was then performed using hematoxylin to stain nuclei and eosin (Wuhan Baiqiandu Biotechnology Co., Ltd., Wuhan, China) to stain the cytoplasm. Finally, the sections were dehydrated through a graded ethanol series, cleared in xylene, and mounted with neutral balsam (Sinopharm Chemical Reagent Co., Ltd., Shanghai, China). The stained sections were examined under a light microscope, and images were captured for analysis. Histological damage to the intestine was scored.

**AB-PAS Staining:** The procedures for tissue fixation, embedding, sectioning, deparaffinization, and rehydration were identical to those described above for HE staining. Following rehydration, sections were stained with Alcian blue by immersing in Alcian blue staining solution (pH 2.5, 1% Alcian blue 8GX in 3% acetic acid; Sigma-Aldrich, St. Louis, MO, USA) at room temperature for 30 min. After rinsing under running water for 5 min, the sections were oxidized with 1% periodic acid aqueous solution (Sinopharm Chemical Reagent Co., Ltd., Shanghai, China) for 10 min, rinsed with distilled water, and then reacted with Schiff’s reagent (Sigma-Aldrich, St. Louis, MO, USA) under light-protected conditions for 20 min. This was followed by a 10-min rinse under running water. Finally, the sections were counterstained with Harris hematoxylin (Sigma-Aldrich, St. Louis, MO, USA) for 1 min, differentiated and blued in running water for 15 min, dehydrated through a graded ethanol series, cleared in xylene, and mounted with neutral balsam. The stained tissue samples were observed under a microscope, and goblet cell numbers were recorded.

### 2.4. Immunohistochemistry (IHC) for Detection of sIgA Distribution

Colon Tissue Collection and Processing: The colon was rapidly excised, rinsed with physiological saline, and fixed in 4% paraformaldehyde for 24 h. The tissues were then paraffin-embedded, sectioned at approximately 4 μm thickness, mounted on slides, and dried.

Immunohistochemistry (IHC) Procedure: Paraffin-embedded colon tissue sections (4 μm thick) were deparaffinized in xylene and rehydrated through a graded ethanol series. Antigen retrieval was performed using citrate buffer (pH 6.0) under high temperature. Subsequently, endogenous peroxidase activity was blocked with 3% hydrogen peroxide, and non-specific binding sites were blocked with 5% bovine serum albumin (BSA). The sections were incubated overnight at 4 °C with a rabbit anti-mouse sIgA primary antibody (1:200 dilution). The following day, after being washed with PBS, the sections were incubated with an HRP-labeled secondary antibody at room temperature for 30 min. Color development was achieved using 3,3′-diaminobenzidine (DAB), followed by counterstaining with hematoxylin. Finally, the sections were dehydrated, cleared, and mounted. The brownish-yellow positive staining signals were observed under a microscope. Multiple fields of view were randomly selected, and ImageJ 1.53c (NIH, Bethesda, MD, USA) was used for quantitative image analysis to evaluate the expression intensity and distribution range of sIgA.

### 2.5. Determination of D-Lactate and DAO Levels

Serum DAO and D-lactate levels were measured using mouse-specific ELISA kits (Jiangsu Meimian Industrial Co., Ltd., Yancheng, China) according to the manufacturer’s instructions. Absorbance was read at 450 nm, and concentrations were calculated from standard curves. All samples were assayed in duplicate.

### 2.6. Determination of Immune Organ Indices

**Immune Organ Indices:** The thymus and spleen were excised, blotted dry to remove surface moisture, and weighed using an electronic balance (ME104E, Mettler Toledo, Columbus, OH, USA). The thymus index and spleen index were calculated as the ratio of the organ weight (mg) to the body weight (g).

Immune Organ Index = Immune Organ Weight (mg)/Body Weight (g)

### 2.7. Measurement of Inflammatory Cytokine Levels by ELISA

Serum levels of TNF-α, IL-6, IL-10, and IL-22 were measured using mouse-specific ELISA kits according to the manufacturer’s instructions. Absorbance was read at 450 nm, and cytokine concentrations (pg/mL) were calculated from standard curves. All samples were assayed in duplicate.

### 2.8. Analysis of Immune Cell Subsets by Flow Cytometry

Analysis of Splenic Immune Cell Populations:

Mouse spleen tissues were gently ground through a 200-mesh cell strainer in PBS to obtain single-cell suspensions. After red blood cell lysis and washing with PBS, cells were treated with an Fc receptor blocking agent for 10 min to prevent non-specific binding.

To analyze helper T cell subsets, intracellular cytokine staining was performed. Cells were first stimulated with a cell stimulation cocktail (containing phorbol ester, ionomycin, and a protein transport inhibitor) in a 37 °C, 5% CO_2_ incubator for 4–6 h. Post-stimulation, cells were stained for surface markers (CD4), followed by fixation and permeabilization using a fixation/permeabilization buffer. Finally, intracellular staining was performed for cytokines (IFN-γ, IL-4, IL-17A) or the transcription factor FOXP3. The specific cell subsets analyzed and their markers were as follows:

Th1 cells: CD4^+^ IFN-γ^+^;

Th2 cells: CD4^+^ IL-4^+^;

Th17 cells: CD4^+^ IL-17A^+^;

Treg cells: CD4^+^ CD25^+^ FOXP3^+^.

After staining with appropriate antibodies, at least 100,000 events per sample were acquired on a NovoCyte 3110 flow cytometer (Agilent, Santa Clara, CA, USA). The percentage of each subset within CD4^+^ T cells was analyzed using FlowJo v10.8.1 (BD Biosciences, San Jose, CA, USA).

### 2.9. Analysis of Gut Microbiota Composition by PacBio 16S Full-Length Sequencing

Total DNA was extracted from colonic contents using the MagBeads FastDNA Kit for Soil (116564384, MP Biomedicals, Santa Ana, CA, USA). The V1-V9 hypervariable regions of the bacterial 16S rRNA gene were amplified by PCR using the primers 27F (5′-Barcode + AGAGTTTGATCMTGGCTCAG-3′) and 1492R (5′-ACCTTGTTACGACTT-3′). Prior to amplification cycles, an initial denaturation step was performed at 98 °C for 25 min in a PCR thermocycler (T100, Bio-Rad, Hercules, CA, USA). Each amplification cycle consisted of denaturation at 98 °C for 30 s, annealing at 56 °C for 30 s, and extension at 72 °C for 45 s. This cycle was repeated 25–30 times, followed by a final extension at 72 °C for 10 min, and then held at 12 °C.

The PCR products were quantified using a microplate reader (Model 680, Beckman Coulter, Brea, CA, USA) and the Quant-iT PicoGreen dsDNA Assay Kit (Invitrogen, Thermo Fisher Scientific, Waltham, MA, USA). Whole-genome and metagenomic libraries were prepared using the SMRTbell^®^ Prep Kit 3.0 (Pacific Biosciences, Menlo Park, CA, USA). After library construction with the SMRTbell^®^ Prep Kit 3.0, circular consensus sequencing (CCS) (version 4.0.0; Pacific Biosciences, Menlo Park, CA, USA) was performed on a PacBio Sequel II system (Pacific Biosciences, Menlo Park, CA, USA). Representative sequences of operational taxonomic units (OTUs) were annotated by BLAST (version 2.2.31; NCBI, Bethesda, MD, USA) against the SILVA_138 database, which contains 16S rRNA sequences of Bacteria and Archaea. Alpha and beta diversity analyses were conducted using QIIME version 1.9.1. Linear discriminant analysis Effect Size (LEfSe) analysis was performed on the online platform at http://huttenhower.sph.harvard.edu/galaxy/, accessed on 10 July 2025. Microbial function was predicted using PICRUSt software (version 1.0.0; Langille et al., 2013), which normalizes species abundance from the raw 16S rRNA sequencing data and maps the species composition data to a functional gene composition table to obtain predicted functional profiles.

### 2.10. Data Processing

All data are expressed as mean ± standard deviation (SD). Normality was assessed using the Shapiro–Wilk test, and homogeneity of variances was assessed using Levene‘s test. For comparisons among all five experimental groups (NC, MC, M, H, and MH), one-way analysis of variance (ANOVA) was performed, followed by Duncan’s multiple range test for post hoc pairwise comparisons. This analysis was applied to DAI scores, immune organ indices, serum cytokine levels (TNF-α, IL-6, IL-10, IL-22), serum DAO and D-lactate levels, goblet cell counts, sIgA expression (MOD), flow cytometry data (Th1, Th2, Th17, Treg proportions), and gut microbiota parameters including alpha diversity (Chao1 index) and relative abundances of key genera (*Lactobacillus*, *Roseburia*, *Prevotella*).

For gut microbiota beta diversity analysis, principal component analysis (PCA) based on unweighted UniFrac distances was performed using QIIME version 1.9.1. Linear discriminant analysis Effect Size (LEfSe) was used to identify differentially abundant microbial taxa among groups, with a threshold of LDA score > 4. For PICRUSt2-predicted metabolic pathways, differences between groups were analyzed using Welch‘s *t*-test, as implemented in the PICRUSt2 pipeline.

A significance threshold of *p* < 0.05 was considered statistically significant. All statistical analyses were performed using SPSS version 27.0 (IBM, Armonk, NY, USA), and graphs were generated using GraphPad Prism version 9.5 (GraphPad Software, San Diego, CA, USA).

## 3. Results and Analysis

### 3.1. HMOs and Alpha-MFGM Ameliorate LPS-Induced Disease Activity and Colonic Histopathological Damage

Hematoxylin-eosin (HE) staining (Figure 1B) revealed structural abnormalities in the mouse intestine. In the MC group, the intestinal architecture was disrupted, and the mucosal layer showed disorganization. Furthermore, substantial inflammatory cell infiltration (indicated by yellow arrows) and extensive cell necrosis characterized by nuclear fragmentation, pyknosis, and hyperchromasia (indicated by red arrows) were observed. Histopathological analysis of the colon (Figure 1E) showed that the MC group had significantly more severe damage than the NC group (*p* < 0.05). In contrast, intestinal pathological damage was markedly ameliorated in the M, H, and MH groups (*p* < 0.05). Notably, the most pronounced improvement was observed in the MH group (*p* < 0.05).

The diarrhea status (Figure 1C) and Disease Activity Index (DAI) (Figure 1D) of the mice were assessed. Compared to the NC group, the MC group exhibited a significant increase in both diarrhea scores and DAI (*p* < 0.05), indicating successful establishment of the LPS-induced model with diarrheal symptoms and overall disease manifestation. Moreover, the diarrhea scores and DAI were significantly reduced in the M, H, and MH groups compared to the MC group (*p* < 0.05). Among the groups, the MH group showed the lowest levels of both diarrhea scores and DAI, with significant differences compared to either the M or H groups alone. These results indicate that both α-MFGM and HMOs can ameliorate pathological injury in mice, and the combination of α-MFGM and HMOs exerts a combined effect and is superior to either α-MFGM or HMOs alone.

### 3.2. HMOs and Alpha-MFGM Ameliorate LPS-Induced Intestinal Immune Injury

**Figure 2:**
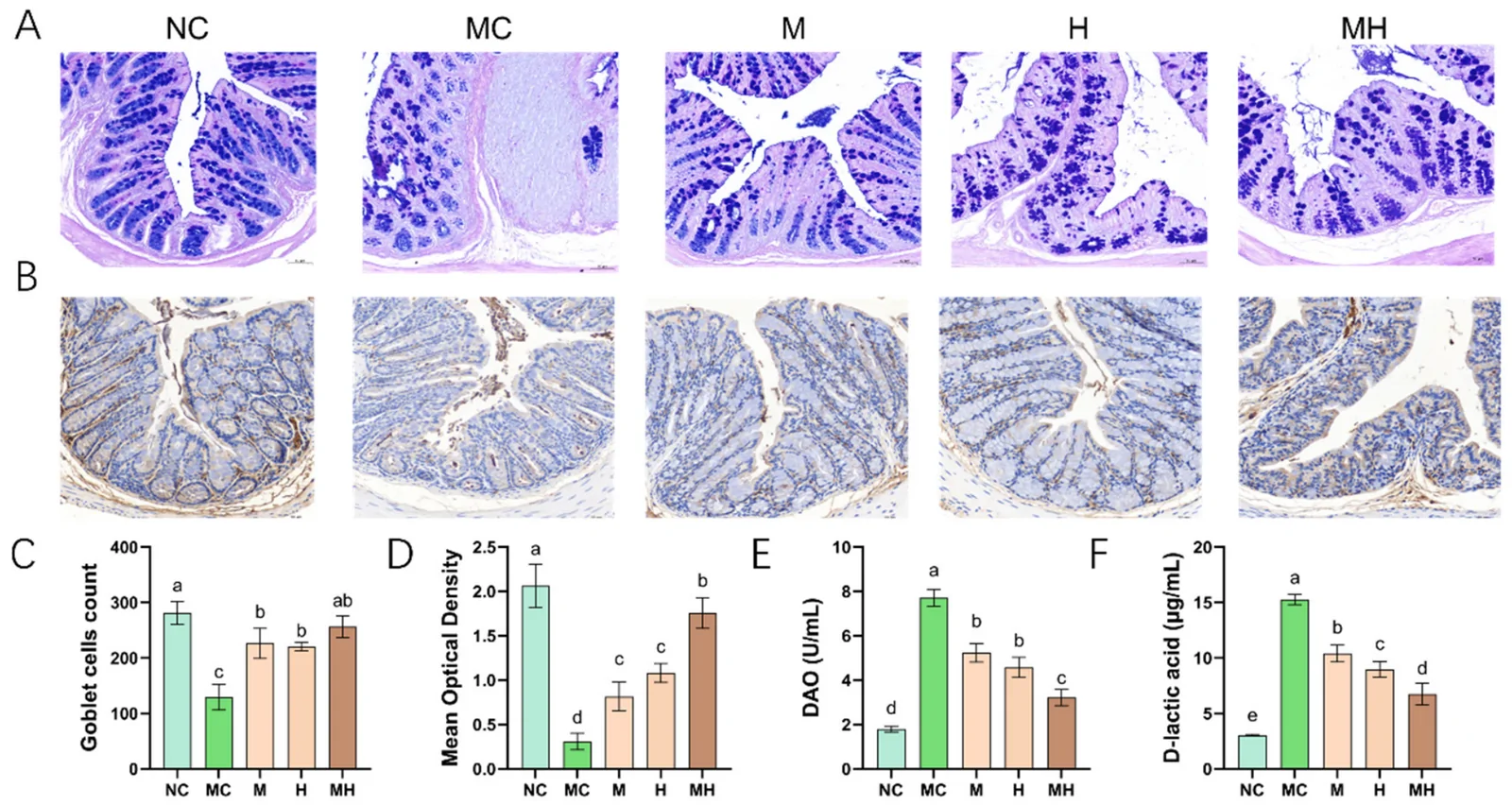
(**A**) AB-PAS staining (200× magnification). (**B**) sIgA immunohistochemistry staining sections (200× magnification). (**C**) Goblet cell count. (**D**) MOD analysis of sIgA expression. (**E**) Serum DAO activity. (**F**) Serum D-lactate concentration. All data are presented as the mean ± SD (*n* = 3). Values with different superscript letters are significantly different at *p* < 0.05.

Alcian Blue-Periodic Acid-Schiff (AB-PAS) staining (Figure 2A) and goblet cell counts (Figure 2C) showed that, compared to the NC group, the number of goblet cells in the colon of MC group mice was significantly reduced (*p* < 0.05), and the mucus layer was thinner, indicating intestinal pathology induced by LPS. Compared to the MC group, the number of goblet cells increased and the mucus layer was partially restored in the M and H groups. In the MH group, the number of goblet cells was markedly increased (*p* < 0.05), and the mucus layer was largely restored to a state close to that of the NC group. This suggests that the combination of α-MFGM and HMOs can effectively promote the recovery of intestinal mucus secretion and enhance mucosal immune capacity in mice.

Representative immunohistochemistry (IHC) sections for secretory IgA (sIgA) are shown in Figure 2B. Quantitative analysis of sIgA-positive staining areas was performed by calculating the Mean Optical Density (MOD) to quantify sIgA expression levels (Figure 2D). Compared to the NC group, sIgA levels were significantly decreased in the MC group (*p* < 0.05), indicating the impact of LPS-induced intestinal injury. sIgA levels were elevated in the M, H, and MH groups, with the MH group showing the most substantial effect, with sIgA levels restored close to those of the NC group (*p* < 0.05). This demonstrates that both α-MFGM and HMOs can significantly enhance plasma cell sIgA secretion and strengthen the intestinal mucosal immune barrier function.

Serum levels of DAO (Figure 2E) and D-lactate (Figure 2F) were significantly elevated in the MC group compared to the NC group (*p* < 0.05). In contrast, serum DAO and D-lactate levels were significantly reduced in the M, H, and MH groups compared to the MC group (*p* < 0.05). Furthermore, the combination of α-MFGM and HMOs resulted in lower DAO and D-lactate levels than those observed with either α-MFGM or HMOs alone. These results indicate that α-MFGM and HMOs can repair the damaged intestinal barrier in mice and that their combination exerts a combined effect, more effectively reducing intestinal permeability and enhancing intestinal barrier function.

### 3.3. HMOs and Alpha-MFGM Improve Systemic Immune Response Levels Induced by LPS

**Figure 3:**
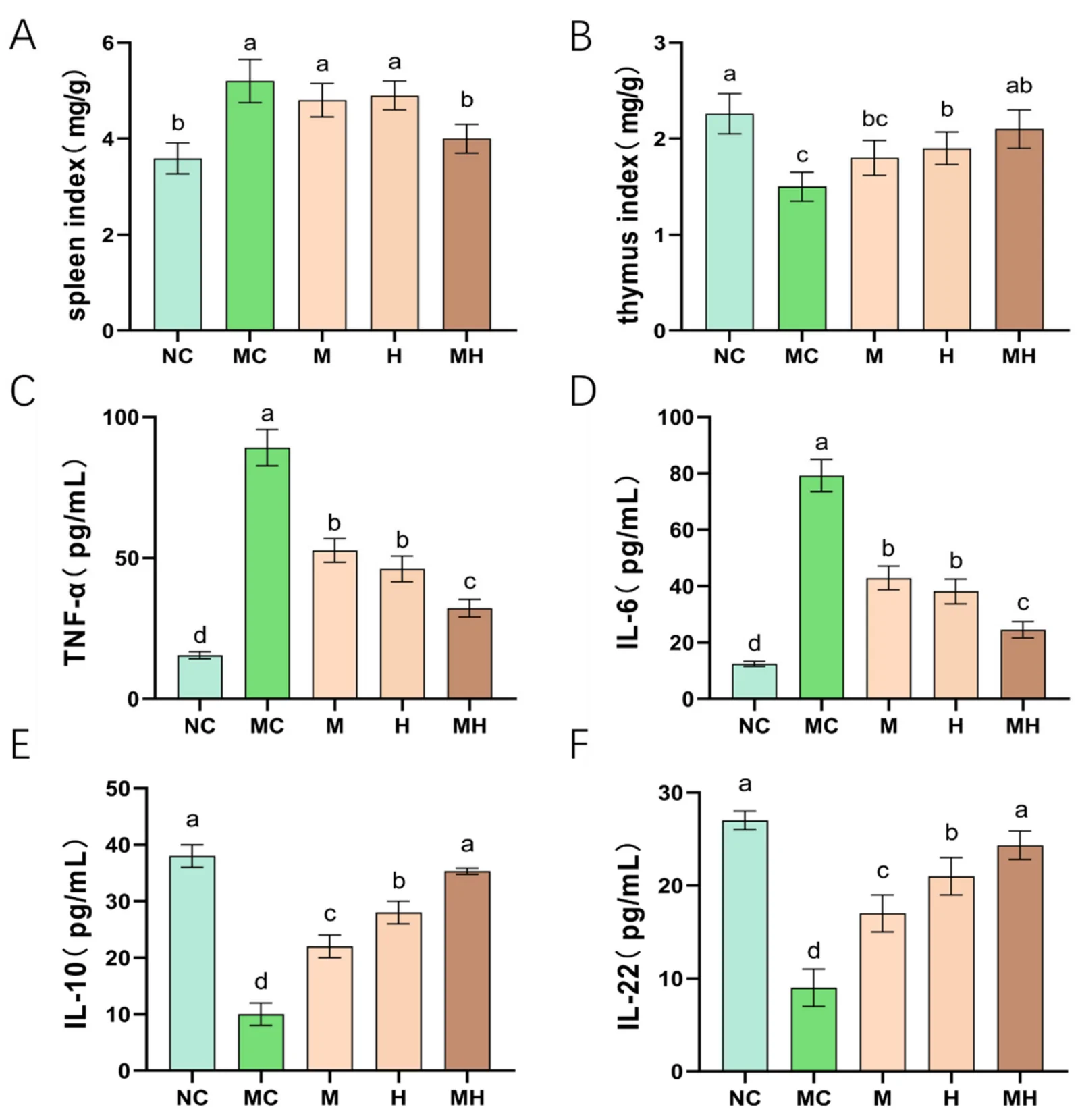
(**A**) Spleen index. (**B**) Thymus index. (**C**) Serum TNF-α level. (**D**) Serum IL-6 level. (**E**) Serum IL-10 level. (**F**) Serum IL-22 level. All data are presented as the mean ± SD (*n* = 3). Values with different superscript letters are significantly different at *p* < 0.05.

As shown in Figure 3A, regarding the spleen index, a significant increase was observed in the MC group compared to the NC group (*p* < 0.05). This is likely because LPS induction triggered an immune response, leading to significant spleen enlargement. Compared to the MC group, the spleen index in the M and H groups showed a decreasing trend but without reaching statistical significance (*p* > 0.05). In contrast, the MH group showed a significant reduction in the spleen index compared to the MC group (*p* < 0.05) and was restored to a normal physiological level, showing no statistical difference from the NC group (*p* > 0.05), indicating that the combination of α-MFGM and HMOs can better modulate the immune response compared to either component alone.

Regarding the thymus index (Figure 3B), it was significantly lower in the MC group than in the NC group (*p* < 0.05). This may be due to LPS-induced immune suppression and thymic atrophy, a crucial immune organ, leading to a decreased thymus index and potentially impaired T-cell development. Compared to the MC group, the thymus index in the M group showed an increasing trend but no significant difference (*p* > 0.05). However, the thymus index increased significantly in both the H and MH groups (*p* < 0.05). Notably, the thymus index in the MH group was not significantly different from that in the NC group (*p* > 0.05), suggesting that the combination of α-MFGM and HMOs better enhances immune capacity and inhibits excessive immune activation under LPS challenge.

The levels of inflammatory cytokines in mouse serum were measured using enzyme-linked immunosorbent assay (ELISA). TNF-α (Figure 3C) is a potent pro-inflammatory cytokine primarily produced by activated macrophages. It activates other inflammatory cells and induces apoptosis. IL-6 (Figure 3D) is a pleiotropic pro-inflammatory cytokine produced by various cells. It is involved in acute-phase responses and immune cell activation (e.g., B cell differentiation into plasma cells). Compared to the NC group, serum levels of the pro-inflammatory factors (TNF-α, IL-6) were significantly elevated in the MC group (*p* < 0.05), indicating that LPS promoted inflammation and disrupted immune function, confirming successful model establishment. In contrast, pro-inflammatory factor levels were significantly reduced in the M, H, and MH groups compared to the MC group (*p* < 0.05). The MH group exhibited the lowest levels of pro-inflammatory cytokines, indicating that both α-MFGM and HMOs can enhance immune function, and their combination has a combined effect, better improving immune function and reducing the production of pro-inflammatory factors.

IL-10 (Figure 3E) is an important anti-inflammatory cytokine mainly produced by regulatory T cells (Tregs), macrophage subsets, and B cells [24]. Its primary functions are to inhibit the production of pro-inflammatory cytokines (e.g., TNF-α, IL-6, IL-1β) and suppress antigen-presenting cell activity, thereby limiting inflammation and promoting immune tolerance and tissue repair. IL-22 (Figure 3F) is an epithelial-protective cytokine involved in barrier repair and antimicrobial defense. Compared to the NC group, the levels of anti-inflammatory factors were significantly lower in the MC group (*p* < 0.05). In contrast, serum levels of anti-inflammatory factors were significantly increased in the M, H, and MH groups compared to the MC group (*p* < 0.05). Particularly in the MH group, anti-inflammatory factor levels were significantly higher than those in the M and H groups (*p* < 0.05) and showed no significant difference from the NC group (*p* > 0.05). This indicates that α-MFGM and HMOs can effectively alleviate LPS-induced diarrheal symptoms and systemic inflammatory responses, enhance the control and repair of inflammatory damage, improve immune function in mice, and increase the production of anti-inflammatory factors.

### 3.4. HMOs and Alpha-MFGM Regulate Immune Cell Subsets

**Figure 4:**
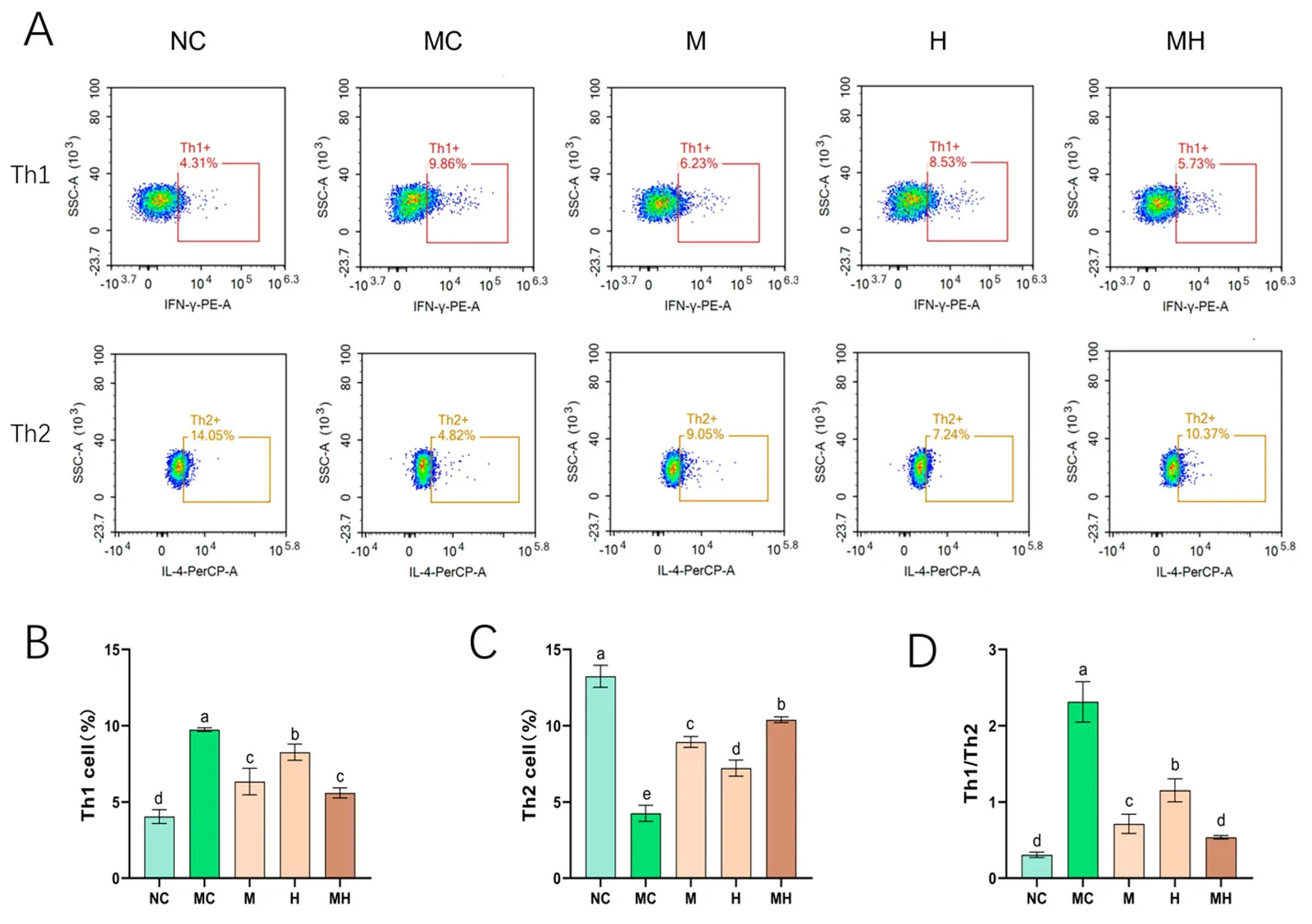
(**A**) Representative flow cytometry plots of Th1 and Th2 cells. (**B**) Proportion of Th1 cells. (**C**) Proportion of Th2 cells. (**D**) Th1/Th2 cell ratio. The color gradient indicates cell density from low (blue) to high (red). The red gate indicates Th1 cells (CD4^+^ IFN-γ^+^), and the yellow gate indicates Th2 cells (CD4^+^ IL-4^+^). All data are presented as the mean ± SD (*n* = 3). Values with different superscript letters are significantly different at *p* < 0.05.

The proportions of helper T cell subsets, namely Th1 and Th2 cells, in the spleen were determined by flow cytometry (Figure 4A). Compared to the NC group, the proportion of Th1 cells (Figure 4B) was significantly increased (*p* < 0.05), while the proportion of Th2 cells (Figure 4C) was significantly decreased (*p* < 0.05) in the MC group. In contrast, compared to the MC group, the proportions of Th1 cells were significantly decreased (*p* < 0.05), and the proportions of Th2 cells were significantly increased (*p* < 0.05) in the M, H, and MH groups. Notably, the ameliorative effects in the M and MH groups were superior to those in the H group. As shown in Figure 4D, the Th1/Th2 cell ratio was significantly elevated in the MC group compared to the NC group following LPS induction (*p* < 0.05). The combined administration of α-MFGM and HMOs reduced the Th1/Th2 ratio compared to the MC group, restoring the ratio comparable to that observed in the NC group. In conjunction with the observed decrease in pro-inflammatory factors like TNF-α and the increase in anti-inflammatory factors like IL-10, these results suggest that α-MFGM/HMOs can suppress LPS-induced excessive Th1 polarization, thereby mitigating inflammatory cytokine-mediated damage to the intestine.

**Figure 5:**
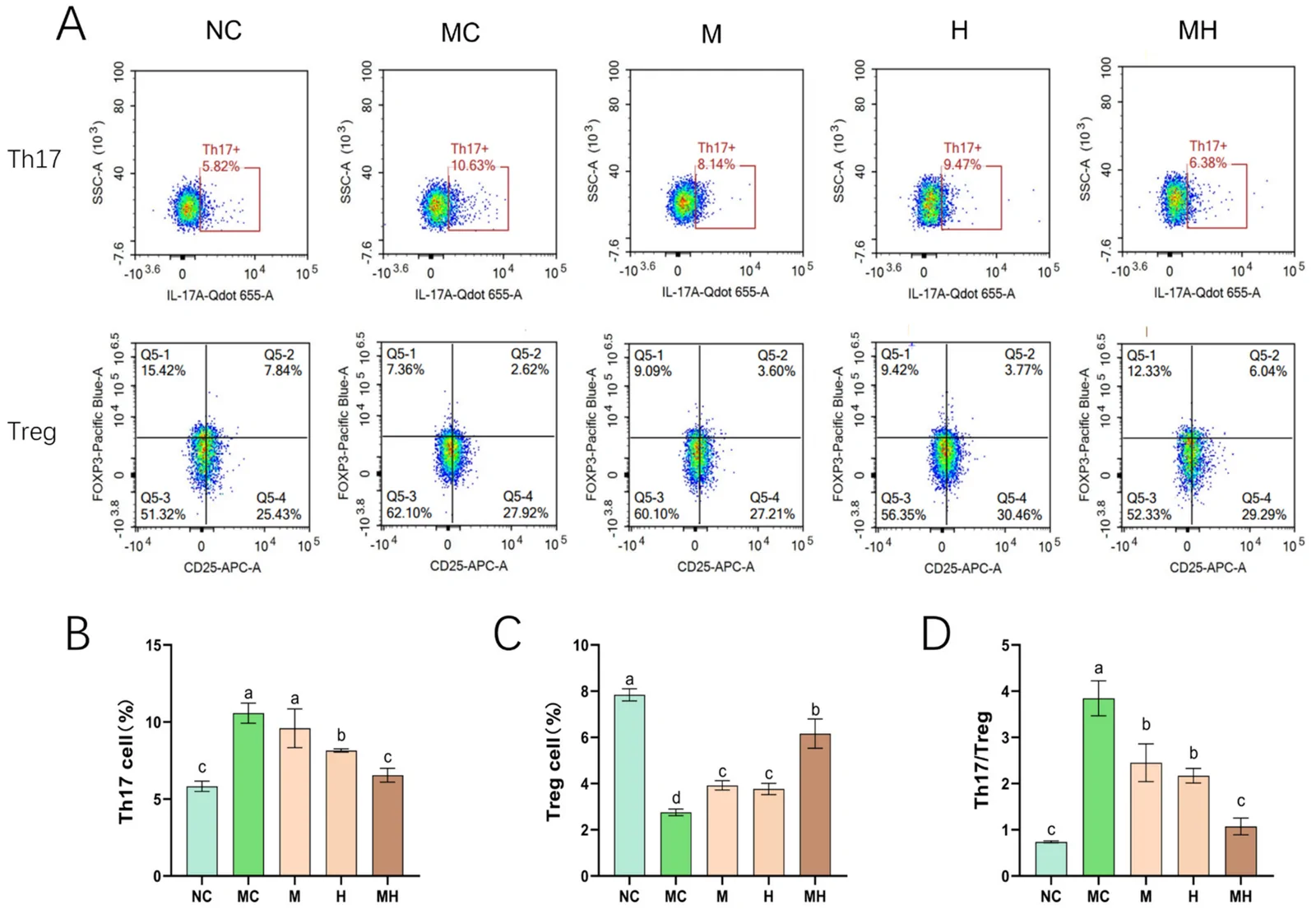
(**A**) Representative flow cytometry plots of Th17 and Treg cells. (**B**) Proportion of Th17 cells. (**C**) Proportion of Treg cells. (**D**) Th17/Treg cell ratio.The color gradient indicates cell density from low (blue) to high (red). The red gate indicates Th17 cells (CD4^+^ IL-17A^+^). In the Treg plots, quadrant Q5-2 represents Treg cells (CD4^+^ CD25^+^ FOXP3^+^). All data are presented as the mean ± SD (*n* = 3). Values with different superscript letters are significantly different at *p* < 0.05.

The proportions of helper T cell subsets, Th17 cells and regulatory T (Treg) cells in the spleen were determined by flow cytometry (Figure 5A). As shown in Figure 5B, compared to the NC group, the proportion of Th17 cells was significantly increased in the MC group (*p* < 0.05), which would exacerbate tissue damage. The combined intervention of α-MFGM and HMOs effectively inhibited this increase, significantly reducing the proportion of Th17 cells compared to the MC group, bringing it close to the normal level observed in the NC group, thereby effectively curbing the inflammatory response.

Regulatory T cells (Tregs) are guardians of intestinal tolerance. As shown in Figure 5C (quadrant Q5-2 representing Treg cells), the proportion of Treg cells was significantly decreased in the MC group compared to the NC group (*p* < 0.05), leading to a collapse of immunosuppressive function. α-MFGM alone significantly increased the Treg cell count (*p* < 0.05), and the combination with HMOs restored it to a level near normal, confirming that the combination of α-MFGM and HMOs helps rebuild the intestinal immune tolerance microenvironment.

The Th17/Treg ratio (Figure 5D) is a key indicator for assessing intestinal immune homeostasis. This ratio was significantly elevated in the MC group compared to the NC group (*p* < 0.05). This shift stemmed from aberrant expansion of Th17 cells and depletion of Treg cells, directly contributing to increased intestinal permeability and inflammatory responses. The combination of α-MFGM and HMOs inhibited Th17 cell differentiation and promoted Treg cell expansion, restoring the ratio to a level not significantly different from that of the NC group (*p* > 0.05). This confirms that the combination of α-MFGM and HMOs can repair the intestinal barrier and alleviate inflammation.

### 3.5. HMOs and Alpha-MFGM Ameliorate LPS-Induced Intestinal Microbial Dysbiosis

**Figure 6:**
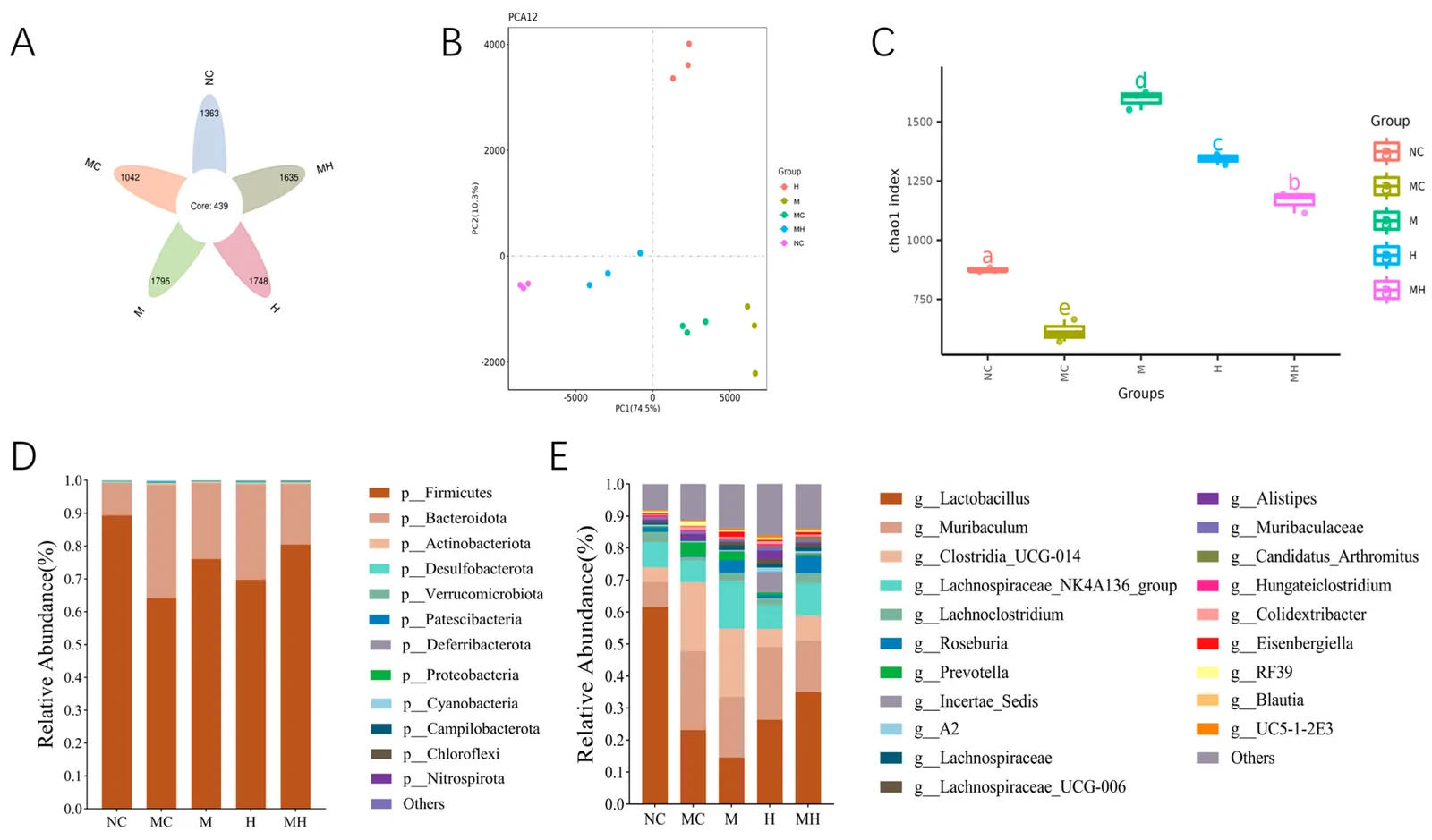
(**A**) Venn diagram. (**B**) Principal Component Analysis (PCA). (**C**) Chao1 index. (**D**) Microbial composition analysis at the phylum level. (**E**) Microbial composition analysis at the genus level. All data are presented as the mean ± SD (*n* = 3). Values with different superscript letters are significantly different at *p* < 0.05.

The Venn diagram (Figure 6A) shows that the NC group reflects the natural diversity and structural specificity of the gut microbial community under normal physiological conditions, representing a complex and stable pattern of a healthy gut microecology. The MC group had the fewest unique operational taxonomic units (OTUs) compared to the NC group, indicating that LPS treatment significantly reduced the diversity of microbial species in the mouse gut. The M, H, and MH groups substantially increased the diversity of microbial communities compared to the MC group. Notably, the microbial diversity in these intervention groups was even higher than that in the NC group, possibly due to the prebiotic effects promoting the colonization of more beneficial bacteria. Principal Component Analysis (PCA) (Figure 6B), a primary method for presenting β-diversity, revealed clear separation among the five groups, indicating distinct overall microbial compositions. The MH group was closest to the NC group in the PCA plot. Analysis of α-diversity showed that the Chao1 index was significantly lower in the MC group than in the NC group (*p* < 0.05), while the Chao1 indices in the M, H, and MH groups were significantly higher than that in the MC group (*p* < 0.05) (Figure 6C). These results indicate that both α-MFGM and HMOs play important roles in modulating the composition of the gut microbiota in mice with LPS-induced intestinal injury.

Further analysis of microbiota changes at the phylum and genus levels showed that at the phylum level, Firmicutes dominated in the NC group, while the abundance of Bacteroidota was relatively lower. LPS treatment decreased the abundance of Firmicutes and increased that of Bacteroidota. Both α-MFGM and HMO interventions reversed this trend (Figure 6D). Analysis at the genus level (Figure 6E) further demonstrated that compared to the MC group, the M group showed an increased abundance of *Roseburia* but a decreased abundance of *Lactobacillus*. Both the H and MH groups exhibited increased abundances of *Lactobacillus* and *Roseburia*, along with a decreased abundance of *Prevotella*. The changes were most pronounced in the MH group, whose microbial profile was closest to that of the NC group. These alterations indicate that α-MFGM and HMOs improved the gut microbiota structure in mice with intestinal injury.

**Figure 7:**
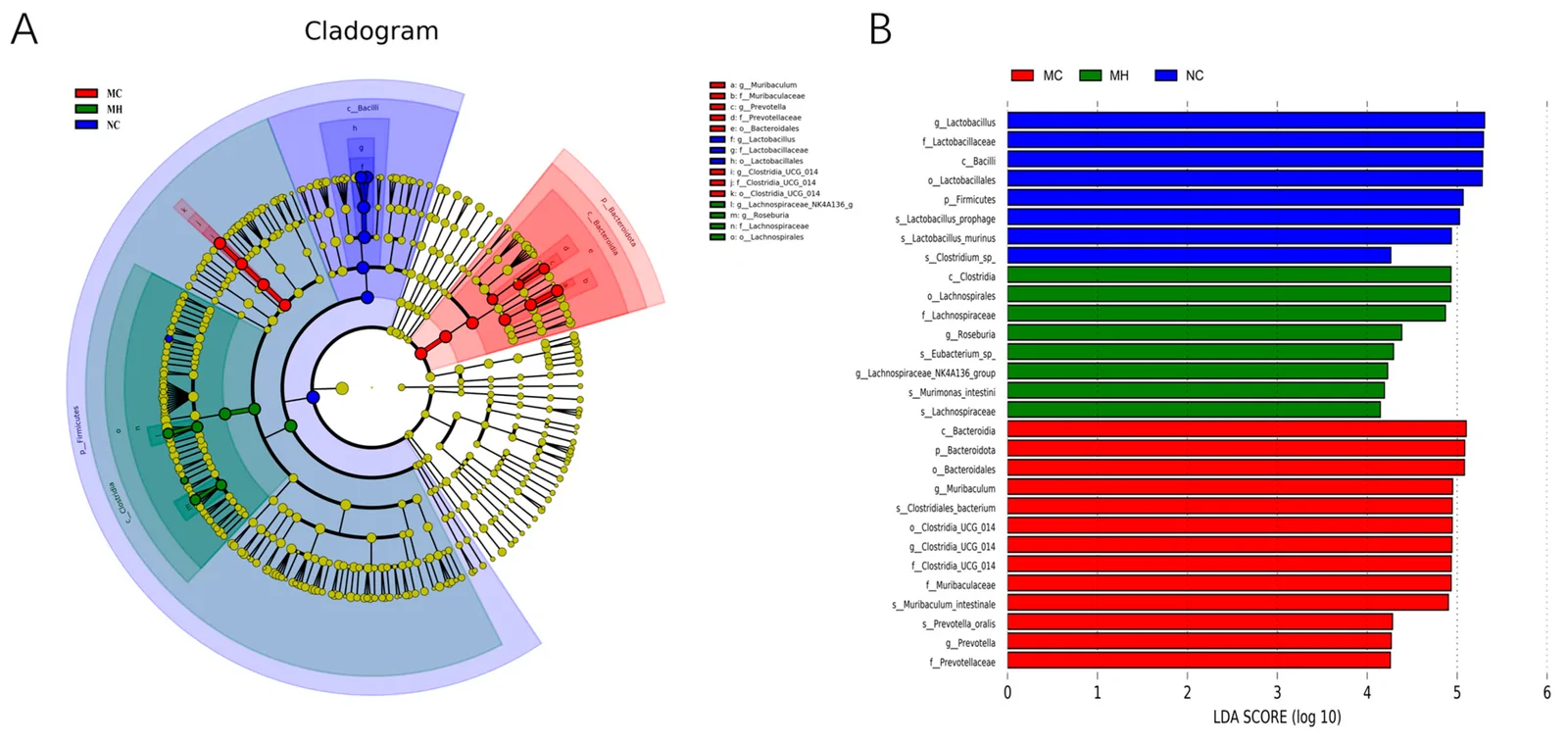
(**A**) Cladogram. (**B**) LDA scores. All data are presented as the mean ± SD (*n* = 3). Values with different superscript letters are significantly different at *p* < 0.05.

Linear discriminant analysis Effect Size (LEfSe) was further employed to identify differentially abundant microbial taxa (biomarkers) among the different treatment groups, with an LDA score > 4 set as the threshold for defining biomarkers (Figure 7B). In this study, we primarily focused on biomarker genera. The results indicated that *Lactobacillus* was the biomarker genus for the NC group, *Roseburia* for the MH group, and *Prevotella* for the MC group. This further substantiates that the combination of α-MFGM and HMOs improves the gut microbiota structure in mice with intestinal injury.

### 3.6. HMOs and Alpha-MFGM Ameliorate LPS-Induced Intestinal Immune Injury by Modulating Microbiota-Related Metabolic Pathways Involved in SCFA Production

**Figure 8:**
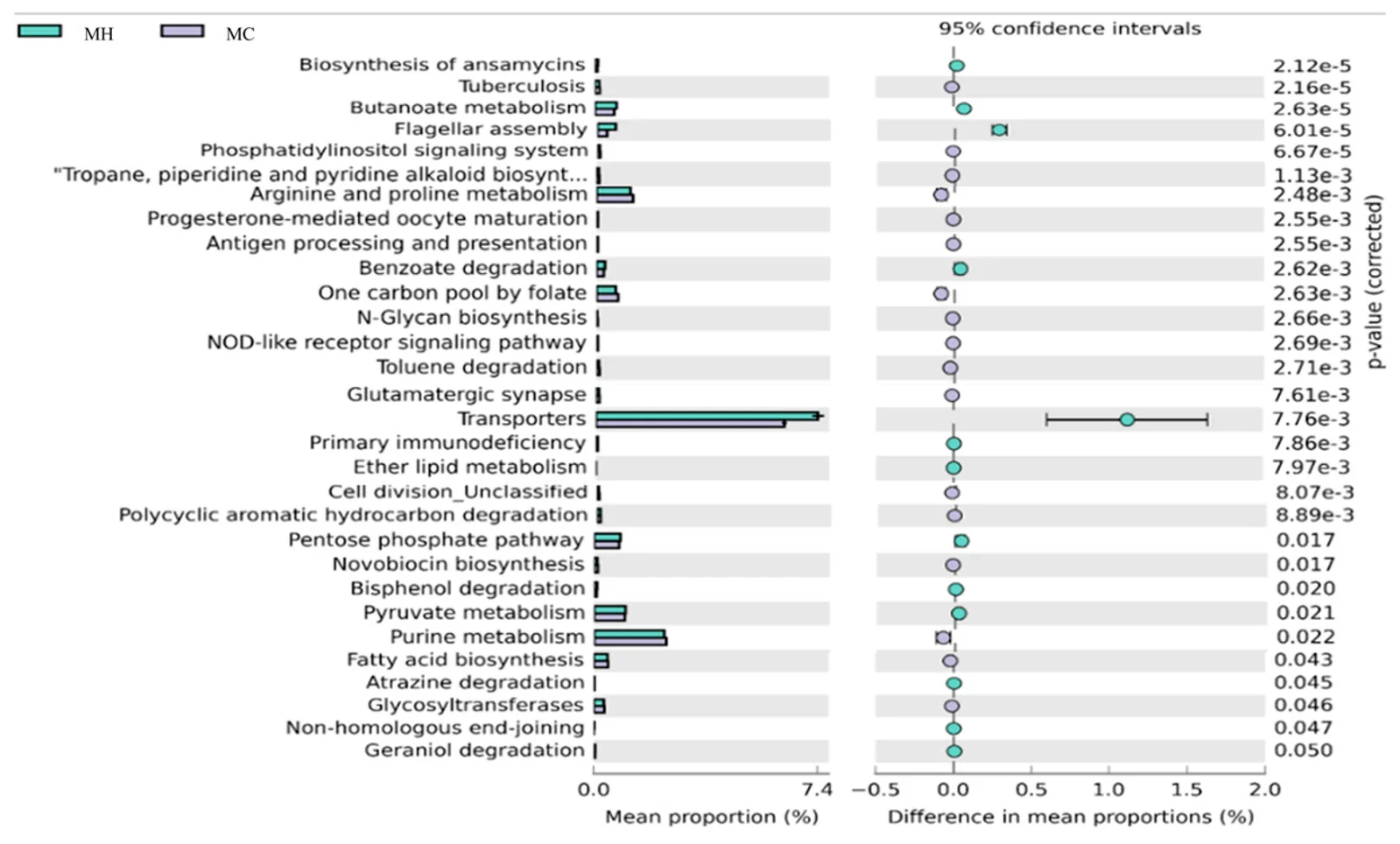
Differential metabolic pathway analysis. All data are presented as the mean ± SD (*n* = 3). Values with different superscript letters are significantly different at *p* < 0.05.

The combined intervention altered the microbiota. To identify the key microbial metabolites mediating immune regulation, we performed functional prediction to pinpoint crucial metabolic pathways and their associated metabolites. As shown in Figure 8, compared to the MC group, the MH group showed significant alterations in several metabolic pathways (Welch’s *t*-test, *p* < 0.05), including Butanoate metabolism, Pyruvate metabolism, and the Pentose phosphate pathway. Butyrate is a core short-chain fatty acid (SCFA), and butanoate metabolism is directly involved in its synthesis, degradation, and metabolic regulation. Pyruvate, the end product of glycolysis, can be converted into SCFAs such as acetate, propionate, and butyrate via microbial fermentation pathways. The Pentose phosphate pathway provides reducing power (NADPH) for microbial fermentation, indirectly promoting SCFA generation. These results demonstrate that α-MFGM and HMOs prevent LPS-induced intestinal mucosal immune injury in mice by modulating the gut microbiota to enhance SCFA production.

## 4. Discussion

The intestine, recognized as the largest immune organ within the human body [2], plays a crucial role in host defense. Promoting the development of the intestinal immune system is vital for reducing susceptibility to infections and diseases. The infant intestinal mucosal immune system serves as a key frontline defense against infection, comprising the mucus layer, epithelial cells, and immune cells [25]. As a critical mucosal barrier in the body, any disruption to mucosal homeostasis can lead to intestinal inflammation [26]. Lipopolysaccharide (LPS), a major component of the outer membrane of Gram-negative bacterial cell walls, can significantly disrupt intestinal epithelial tight junctions after injection or intraperitoneal administration by activating the host’s TLR4-dependent signaling pathway [27]. Previous studies have shown that either HMOs or MFGM alone can effectively ameliorate intestinal mucosal immune injury [28,29]. However, research investigating the combined effects of their combination on improving intestinal mucosal immune injury is lacking. Therefore, in this study, we established an intestinal immune injury model in mice via intraperitoneal injection of LPS to study LPS-induced intestinal mucosal damage. This study aimed to evaluate the combined effects of HMOs and α-MFGM on intestinal mucosal and systemic immune dysfunction in an LPS-induced mouse model and to explore the role of gut microbiota modulation in this process.

We found that LPS had a substantial impact on the integrity of the intestinal mucosal structure in mice, which is consistent with previous research findings [30]. In the LPS group, we observed abnormal intestinal tissue architecture and disorganization of the mucosal layer. The tissue exhibited substantial inflammatory cell infiltration, extensive cell necrosis, nuclear fragmentation, and pyknosis with hyperchromasia. In contrast, in the group receiving the combination of HMOs and α-MFGM, the intestinal tissue structure showed only mild abnormalities. Within the field of view, the epithelial cells of the mucosal layer were tightly arranged without detachment, crypt structures were intact and orderly, the submucosa showed no edema, and only occasional inflammatory cell infiltration was observed. This aligns with the findings of Chen et al. [31]. Furthermore, we quantified the pathological condition of the mice using diarrhea scores and the Disease Activity Index (DAI). Based on the above findings, both HMOs or α-MFGM alone could effectively alleviate acute inflammation-induced intestinal mucosal immune injury, which is consistent with existing studies [12,29]. However, we found that their combination yielded the best results, demonstrating a significant combined effect.

Goblet cells on the mucosal surface produce mucus, which serves as a primary barrier limiting contact between the host and commensal flora and preventing microbial translocation [32]. We observed a reduction in the number of goblet cells and damage to the intestinal barrier in the LPS group, which aligns with previous research findings [33]. In the current study, HMOs alone significantly increased the number of goblet cells, thereby enhancing mucosal immune capacity. Furthermore, we observed that the combination of HMOs and α-MFGM yielded an even better effect. Secretory IgA (sIgA) in the intestine prevents pathogen adhesion and entry into the intestinal barrier and also modulates the composition of the gut microbiota, contributing to intestinal homeostasis [34]. Our study found that the combination of HMOs and α-MFGM significantly increased the expression of sIgA in the intestine.

Concurrently, studies have shown that diamine oxidase (DAO) is primarily located in the cytoplasm of mature intestinal epithelial cells at the tips of small intestinal mucosal villi. When the intestinal mucosa is damaged and permeability increases (“leaky gut”), DAO is released in large quantities into the bloodstream, leading to elevated serum DAO activity. D-lactate is mainly produced by fermentation of intestinal bacteria (particularly Gram-positive and anaerobic bacteria). When intestinal permeability increases significantly, D-lactate produced in the gut can cross the damaged intestinal mucosal barrier and enter the systemic circulation [35]. This is consistent with our findings. The combination of HMOs and α-MFGM effectively reduced intestinal permeability.

Simultaneously, after intestinal mucosal immune injury occurs, it can further affect systemic immune responses. The spleen serves as a major reservoir for immune cells in mammals. Various stimuli can disrupt splenic homeostasis, leading to spleen damage and immune dysfunction [36]; Concurrently, the thymus is an important primary immune organ that is susceptible to atrophy upon exposure to toxins [37] In our study, we found that after LPS stimulation, the spleen index increased and the thymus index decreased in mice, which is consistent with the findings of Sun et al. [38]. After the combined administration of HMOs and α-MFGM, the spleen index was reduced while the thymus index was upregulated, achieving a dynamic balance in the spleen/thymus indices. This alleviated the inflammatory condition in mice and achieved modulation of the systemic immune response.

LPS is present in the outer membrane of Gram-negative bacteria [39], it stimulates macrophages to secrete pro-inflammatory cytokines, including IL-6 and TNF-α, and promotes the synthesis and release of inflammatory cytokines. Previous studies have shown that HMOs can alleviate LPS-induced intestinal inflammation by reducing the secretion of pro-inflammatory factors while increasing the secretion of anti-inflammatory factors [12]. Similarly, studies have confirmed that oral administration of MFGM to low-body-weight mice can also alleviate LPS-induced intestinal inflammation and reduce the secretion of pro-inflammatory factors such as TNF-α and IL-6 [40]. This aligns with our current findings. Moreover, we observed that their combination resulted in more pronounced effects, demonstrating a clear combined action.

Following LPS stimulation, the imbalance of immune cell subsets is further exacerbated, particularly affecting helper T cell subsets. Th1 cells orchestrate cell-mediated immunity by secreting cytokines such as IFN-γ and TNF-α, promoting macrophage activation and the killing of intracellular pathogens (e.g., bacteria, viruses). However, over-activation can trigger intense inflammatory responses, leading to tissue damage. Th2 cells govern humoral immunity by secreting cytokines like IL-4, IL-5, and IL-13, mediating antibody production and anti-parasitic immunity, while also possessing anti-inflammatory and immunomodulatory functions [41]. Under normal physiological conditions, Th1/Th2 cells maintain a dynamic balance [42]. Our results indicate that the combination of HMOs and α-MFGM ameliorated the LPS-induced Th1/Th2 imbalance, suppressing the abnormal expansion of Th1 cells and promoting the recovery of Th2 cells. This aligns with the findings reported by Dong et al. [33].

Pro-inflammatory Th17 cells undergo aberrant expansion in inflammatory models, and the IL-17A they secrete directly compromises epithelial barrier integrity [43]. Regulatory T cells (Tregs) play a pivotal role in immunosuppression and tolerance, protecting the body from autoimmunity and inflammation [44]. Recent studies have suggested an association between Th17/Treg imbalance and inflammatory responses [45]. Based on this, we observed a significant increase in the proportion of Th17 cells and a concurrent significant decrease in the proportion of Treg cells in the LPS group. This is consistent with the previous findings of Zhang et al., who studied the combination of HMOs and osteopontin, an active component within α-MFGM [46]. HMOs alone can effectively alleviate this phenomenon by inhibiting Th17 cell differentiation and promoting Treg cell expansion. This is consistent with the results we observed using the combination of HMOs and α-MFGM, and furthermore, the combination demonstrated superior efficacy compared to using HMOs alone.

The gut microbiota exerts a substantial influence on the regulation of intestinal mucosal immunity [47]. Both HMOs and MFGM have been confirmed to effectively modulate the composition of the gut microbiota, thereby mitigating the dysbiosis caused by LPS. Among the gut microbiota, *Firmicutes* and *Bacteroidota* predominate [48]. In our study, we observed that LPS treatment led to a decrease in *Firmicutes* and an increase in *Bacteroidota*, which also indicates the significant role these phyla play in immune regulation. This observation is consistent with previous research findings [46].

*Lactobacillus* is a common beneficial bacterium in the gut. Numerous studies have shown its important impact on maintaining intestinal health [49]. It can alleviate LPS-induced intestinal barrier damage through its metabolites. The study by Zhang et al. showed that the combination of HMOs and osteopontin (a component of α-MFGM) ameliorated the LPS-induced reduction in *Lactobacillus* abundance and increased its levels [46]. This aligns with our observations. *Roseburia* is a classic butyrate-producing bacterium. Its metabolite, butyrate, is widely recognized as a crucial energy source for the intestinal mucosa and also possesses anti-inflammatory and barrier-repairing properties [50]. The combination of HMOs and α-MFGM significantly increased its abundance.

*Prevotella* is a genus of anaerobic bacteria commonly found in the guts of various hosts. Its abundance is significantly influenced by dietary structure, particularly high-fiber and high-carbohydrate diets. Under certain conditions, it can produce beneficial fermentation products. However, in an inflammatory context or under conditions of dysbiosis, its biological role often skews towards being pro-inflammatory and damaging to mucosal homeostasis [51]. In the intestines of LPS-treated mice, we observed a significant increase in the abundance of *Prevotella*. The combination of HMOs and α-MFGM significantly reversed this trend.

The combined intervention altered the microbiota. Through functional prediction, we further investigated the key metabolic pathways through which HMOs and α-MFGM improve microbiota-mediated immune regulation. We found that HMOs and α-MFGM primarily modulate immunity by regulating the microbial production of SCFAs. SCFAs, mainly including acetate, propionate, and butyrate, are major metabolites derived from microbial fermentation of dietary fiber and are considered crucial mediators for regulating mucosal immunity and intestinal homeostasis [52]. Current research confirms that SCFAs have a protective effect against LPS-induced inflammation by inhibiting the NF-κB pathway and reducing the production of inflammatory factors [53]. Therefore, we speculate that HMOs and α-MFGM may regulate immunity by modulating the gut microbiota and enhancing the production of SCFAs. However, SCFA concentrations were not directly measured, and this mechanism requires future verification.

The integrated analysis of gut microbiota and functional predictions offers a plausible mechanistic framework: the combined intervention enriched butyrate-producing genera (*Lactobacillus* and *Roseburia*) and reduced *Prevotella* abundance, paralleled by PICRUSt2-predicted enhancement of butanoate metabolism. We hypothesize that this microbiota shift may contribute to the observed improvements in barrier function and immune balance, but SCFA concentrations were not directly measured and causality remains to be established. Despite these limitations—acute LPS model with 6-h observation, functional predictions rather than direct metabolite measurements, and endpoint microbiota analysis—our findings provide a basis for further mechanistic and translational research.

## 5. Conclusions

This study systematically elucidated the combined protective effects of human milk oligosaccharides (HMOs) and α-milk fat globule membrane (α-MFGM) against lipopolysaccharide (LPS)-induced intestinal immune injury in mice and explored the underlying mechanisms. The results demonstrate that the combined intervention of HMOs and α-MFGM, compared to individual components, more effectively ameliorated intestinal histopathological damage, restored goblet cell numbers and mucus layer thickness, enhanced the mucosal immune response of secretory immunoglobulin A (sIgA), and significantly reduced serum D-lactate and diamine oxidase (DAO) levels. This indicates a combined effect in repairing both the physical and immune barrier functions of the intestine. At the systemic immunomodulatory level, the combined intervention significantly balanced the spleen and thymus indices and effectively regulated the inflammatory response, manifested by decreased levels of pro-inflammatory factors (TNF-α, IL-6) and increased levels of IL-10, an immunoregulatory cytokine, and IL-22, an epithelial-protective cytokine involved in barrier repair and antimicrobial defense. At the cellular immunity level, the combined intervention significantly corrected the LPS-induced imbalance of helper T cell subsets: it suppressed the excessive polarization of pro-inflammatory Th1 cells and the differentiation of Th17 cells while simultaneously promoting the Th2-type immune response and the proliferation of regulatory T cells (Tregs). Consequently, the key ratios of Th1/Th2 and Th17/Treg were restored to balance, reconstructing intestinal immune homeostasis. This study further unraveled the mechanism of action from the perspective of microbiota-host interactions. The combined use of HMOs and α-MFGM significantly reversed LPS-induced gut dysbiosis and optimized the microbial structure, specifically by increasing the abundance of beneficial bacteria (e.g., *Lactobacillus* and *Roseburia*) and suppressing the proliferation of potentially pathogenic bacteria (e.g., *Prevotella*). Functional prediction analysis revealed that this combined protection is closely associated with the modulation of short-chain fatty acid (SCFA) synthesis pathways, such as butanoate metabolism, with SCFAs playing a central role as key immunomodulatory metabolites. In summary, this study reveals that HMOs and α-MFGM exert combined effects through a connected pathway: modulating gut microbiota structure, promoting the production of microbial metabolites (SCFAs), enhancing intestinal physical and immune barrier functions, and ultimately balancing local and systemic immune responses, thereby jointly safeguarding intestinal mucosal immune health. This research provides new scientific evidence for a deeper understanding of the combined functions of key bioactive components in human milk and offers potential experimental basis and development directions for creating innovative infant formula with immune-supportive functions.

## Figures and Tables

**Table 1:** Hart and Dobb Diarrhea Scoring Criteria.

Score	Description of Fecal State
0	Normal, dry, and formed feces
1	Mildly soft but still formed
2	Obviously moist, unformed, with slight adhesion to the anus or tail
3	Loose, watery feces, causing obvious perianal soiling
4	Severe watery diarrhea, containing copious mucus or blood, accompanied by obvious perianal fur soiling or hair loss

**Table 2:** Disease Activity Index (DAI) Scoring Details.

Item	Scoring Criterion	0 Points	1 Point	2 Points	3 Points	4 Points
**Weight Change**	Percentage change compared to Day 1	No change or increase	↓1–5%	↓5–10%	↓10–15%	>15% decrease
**Stool Consistency**	Fecal appearance and morphology	Normal	Slightly soft	Paste-like, unformed	Watery or containing blood/mucus	---
**Fecal Blood**	Presence of blood in feces (gross/occult)	None	Slight occult blood	Grossly bloody stool	Grossly bloody stool with reduced activity	---

DAI calculation: DAI = (Body weight change score) + (Stool consistency score) + (Fecal blood score). The DAI was used for intergroup comparative analysis. “↓” indicates a decrease; “---” indicates not applicable.

## Data Availability

The raw data supporting the conclusions of this article will be made available by the authors on request.
